# Extraembryonic tissues: exploring concepts, definitions and functions across the animal kingdom

**DOI:** 10.1098/rstb.2021.0250

**Published:** 2022-12-05

**Authors:** Guojun Sheng, Thorsten E. Boroviak, Urs Schmidt-Ott, Shankar Srinivas

**Affiliations:** ^1^ International Research Center for Medical Sciences, Kumamoto University, Kumamoto 860-0811, Japan; ^2^ Department of Physiology, Development and Neuroscience, University of Cambridge, Cambridge CB2 3EG, UK; ^3^ Department of Organismal Biology and Anatomy, University of Chicago, Chicago, IL 60637, USA; ^4^ Department of Physiology, Anatomy and Genetics, University of Oxford, Oxford OX3 7TY, UK

## 1. Introduction

All emerging life must function while being built. The developing embryo requires an aqueous environment, a supply of nutrients, gas exchange and waste management. Extraembryonic membranes accomplish these challenges by providing multifunctional organs and interfaces between the embryo and the outside world.

The importance of extraembryonic tissues is particularly evident in animals that have adapted to terrestrial life. For instance, amniotic vertebrates have four extraembryonic tissues: amnion, chorion, allantois and yolk sac, with each performing specialized, essential physiological functions. Terrestrial arthropods have evolved other extraembryonic tissues, such as the serosa and amnion of insects, which perform similar functions to their vertebrate counterparts (e.g. in drought resistance, embryonic tissue morphogenesis and possibly waste management) and more (e.g. in innate immunity). Traditionally, extraembryonic tissues were perceived to function only during embryogenesis, subsequently being shed at birth or hatching.

Under close scrutiny, however, this embryonic–extraembryonic dichotomy is far from being straight-forward. The boundaries of embryonic and extraembryonic tissues are often ill-defined; the fates of the so-called extraembryonic tissues may not be entirely extraembryonic; their physiological, biomechanical, morphogenetic and signalling roles, with regard to embryonic development are under-appreciated; and evolutionary constraints on conservation and diversification of extraembryonic tissues are poorly understood. Increasingly, molecular and cellular evidence points toward certain arbitrariness in making such a distinction between embryonic and extraembryonic tissues. Therefore, we feel it is timely to dedicate a theme issue to the current understanding of cellular entities that are traditionally viewed as extraembryonic.

Our goal in this theme issue is to present evidence for and raise awareness of both the interconnection between the embryonic and extraembryonic tissues, as well as the importance of further studies for a ‘holistic' understanding of animal development.

This issue features review and research articles on extraembryonic tissue complements in vertebrates and panarthropods. Among them, eight articles focus on mammalian extraembryonic tissues. Matsuo *et al*. [1] discuss developmental and mechanical roles of Reichert's membrane in mice, a non-cellular basal lamina separating trophectoderm and parietal yolk sac endoderm. Thowfeequ & Srinivas [2] present the idea of shifting boundaries between embryonic and extraembryonic tissues in time and space during early mouse development. Pfeffer [3] hypothesizes a connection between cavitation of the epiblast and loss of Rauber's layer (polar trophoblast covering the inner cell mass) in mammals. Chowdhary & Hadjantonakis [4] describe the ontogeny and specification of primitive endoderm in mice. Downs [5] examines roles of the allantois during embryogenesis in mice. Siriwardena & Boroviak [6] compare embryo implantation in different primate species and suggest that the mechanisms controlling invasion depth may provide new insights into human placental disorders. Chuva de Sousa Lopes *et al*. [7] review modes of amniogenesis in amniotes and morphogenetic and molecular signatures of amniotic ectoderm and mesoderm in mice. Roelen & Chuva de Sousa Lopes [8] discuss primordial germ cell specification at the boundary of embryonic and extraembryonic territories and their subsequent gonadal colonization.

Extraembryonic tissues in non-mammalian vertebrates are the focus of four additional papers. Concha & Reig [9] give a comprehensive overview of the origin, form, and function of teleost extraembryonic structures, that is, the yolk syncytial layer and the enveloping layer. Wen *et al*. [10] investigate differences in molecular signatures of the chorioallantoic membrane between the live-bearing and egg-laying lizards. Nagai *et al*. [11] describe morphogenesis of the chorioallantoic membrane in chick development and hypothesize an evolutionarily conserved process underlying extraembryonic mesothelial fusion in chorioallantoic membrane formation in amniotes and chorioallantoic placenta formation in eutherian mammals. Wittington *et al*. [12] compare placentation across vertebrates, reminding readers of the deep evolutionary roots of placentation and of diverse strategies adopted in different vertebrate groups to fulfill similar physiological needs.

Extraembryonic tissues in the Panarthropoda are discussed in five articles. Panfilio & Chuva de Sousa Lopes [13] compare the anatomy, cellular ontogeny, physiological function, evolutionary roles, molecular signatures and genetic regulation between amniotes and insects. Treffkorn *et al*. [14] assess the potential occurrence of extraembryonic tissues in onychophorans (velvet worms) and tardigrades (water bears), two invertebrate clades that are closely related to arthropods. Prpic & Pechmann [15] discuss candidate extraembryonic tissues in spiders and other chelicerates. Jacob *et al*. [16] provide evidence for an immune function of the serosa in hemimetabolous insects, which previously was only known from holometabolous insects. Some flies, including the fruit fly *Drosophila melanogaster* lack a differentiated serosa along with its immune function, and instead develop a unique extraembryonic tissue, dubbed amnioserosa. Schmidt-Ott & Kwan [17] trace the serosa and amnion heritage of this tissue and examine how the serosa-specific and amnio-specific developmental trajectories have merged into one in *D. melanogaster*.

These articles offer snapshot views of both the intricacy and complexity of embryonic-extraembryonic crosstalk in animal development. However, in contrast to embryonic development, in which concepts such as gastrulation, germ layer formation, and dorsal-ventral and anterior–posterior axis formation can be broadly applied to all metazoans, no such unifying themes have emerged for extraembryonic tissue development. We hope that this theme issue will encourage more biologists to work on extraembryonic tissues, investigating questions regarding their roles in animal phylogeny and ontogeny and their potential application in regenerative medicine. For example, are there common defining features and functions of extraembryonic tissues across vertebrates and invertebrates? Do extraembryonic tissues follow unique evolutionary trajectories that are largely decoupled from embryonic tissues to meet specific physiological needs present for different organisms during development? Which extraembryonic tissues in vertebrates and panarthropods contribute cells to the fetus and what are the fates of these cells? How are extraembryonic tissues involved in embryonic patterning? Do the so-called extraembryonic tissues of onychophorans and chelicerates contribute lasting embryonic tissue? What is the function of insect ventral amnion (and amniotic cavity)? What drove its loss in cyclorrhaphan flies? Does the residual (dorsal) amnion of lower Cyclorrhapha drive the same morphogenetic movements than the amnioserosa? If so, how does the dorsal amnion power these processes?

Finally, a more practical use of knowledge of extraembryonic tissues will be to model mammalian development *in vitro* by reconstituting the physiological environment of the fetal–maternal interface, a role usually fulfilled by extraembryonic tissues. Currently, we know relatively little about molecular and morphogenetic principles by which we can guide the differentiation of embryonic stem cells towards proper organization of any extraembryonic tissue. We hope this theme issue will spark discussions, debate and collaborations within the field, ultimately moving research on these essential but still somewhat mysterious tissues to a new level.

## Data Availability

This article has no additional data.

## References

[1] Matsuo I, Kimura-Yoshida C, Ueda Y. 2022 Developmental and mechanical roles of Reichert's membrane in mouse embryos. Phil. Trans. R. Soc. B **377**, 20210257. (10.1098/rstb.2021.0257) PMC9574627 36252218

[2] Thowfeequ S, Srinivas S. 2022 Embryonic and extraembryonic tissues during mammalian development: shifting boundaries in time and space. Phil. Trans. R. Soc. B **377**, 20210255. (10.1098/rstb.2021.0255) PMC9574638 36252217

[3] Pfeffer PL. 2022 Alternative mammalian strategies leading towards gastrulation: losing polar trophoblast (Rauber's layer) or gaining an epiblast cavity. Phil. Trans. R. Soc. B **377**, 20210254. (10.1098/rstb.2021.0254) PMC9574635 36252216

[4] Chowdhary S, Hadjantonakis A-K. 2022 Journey of the mouse primitive endoderm: from specification to maturation. Phil. Trans. R. Soc. B **377**, 20210252. (10.1098/rstb.2021.0252) PMC9574636 36252215

[5] Downs KM. 2022 The mouse allantois: new insights at the embryonic–extraembryonic interface. Phil. Trans. R. Soc. B **377**, 20210251. (10.1098/rstb.2021.0251) PMC9574631 36252214

[6] Siriwardena D, Boroviak TE. 2022 Evolutionary divergence of embryo implantation in primates. Phil. Trans. R. Soc. B **377**, 20210256. (10.1098/rstb.2021.0256) PMC9574630 36252209

[7] Chuva de Sousa Lopes SM, Roelen BAJ, Lawson KA, Zwijsen A. 2022 The development of the amnion in mice and other amniotes. Phil. Trans. R. Soc. B **377**, 20210258. (10.1098/rstb.2021.0258) PMC9574641 36252226

[8] Roelen BAJ, Chuva de Sousa Lopes SM. 2022 Stay on the road: from germ cell specification to gonadal colonization in mammals. Phil. Trans. R. Soc. B **377**, 20210259. (10.1098/rstb.2021.0259) PMC9574628 36252219

[9] Concha ML, Reig G. 2022 Origin, form and function of extraembryonic structures in teleost fishes. Phil. Trans. R. Soc. B **377**, 20210264. (10.1098/rstb.2021.0264) PMC9574637 36252221

[10] Wen J, Ishihara T, Renfree MB, Griffith OW. 2022 Comparing the potential for maternal–fetal signalling in oviparous and viviparous lizards. Phil. Trans. R. Soc. B **377**, 20210262. (10.1098/rstb.2021.0262) PMC9574625 36252210

[11] Nagai H, Tanoue Y, Nakamura T, Chan CJJ, Yamada S, Saitou M, Fukuda T, Sheng G. 2022 Mesothelial fusion mediates chorioallantoic membrane formation. Phil. Trans. R. Soc. B **377**, 20210263. (10.1098/rstb.2021.0263) PMC9574633 36252211

[12] Whittington CM, Buddle AL, Griffith OW, Carter AM. 2022 Embryonic specializations for vertebrate placentation. Phil. Trans. R. Soc. B **377**, 20210261. (10.1098/rstb.2021.0261) PMC9574634 36252220

[13] Panfilio KA, Chuva de Sousa Lopes SM. 2022 The extended analogy of extraembryonic development in insects and amniotes. Phil. Trans. R. Soc. B **377**, 20210268. (10.1098/rstb.2021.0268) PMC9574626 36252225

[14] Treffkorn S, Mayer G, Janssen R. 2022 Review of extra-embryonic tissues in the closest arthropod relatives, onychophorans and tardigrades. Phil. Trans. R. Soc. B **377**, 20210270. (10.1098/rstb.2021.0270) PMC9574629 36252224

[15] Prpic N-M, Pechmann M. 2022 Extraembryonic tissue in chelicerates: a review and outlook. Phil. Trans. R. Soc. B **377**, 20210269. (10.1098/rstb.2021.0269) PMC9574639 36252223

[16] Jacobs CGC, van der Hulst R, Chen Y-T, Williamson RP, Roth S, van der Zee M. 2022 Immune function of the serosa in hemimetabolous insect eggs. Phil. Trans. R. Soc. B **377**, 20210266. (10.1098/rstb.2021.0266) PMC9574632 36252212

[17] Schmidt-Ott U, Kwan CW. 2022 How two extraembryonic epithelia became one: serosa and amnion features and functions of *Drosophila*'s amnioserosa. Phil. Trans. R. Soc. B **377**, 20210265. (10.1098/rstb.2021.0265) PMC9574642 36252222

